# Multifunctional Electrically Conductive Copper Electroplated Fabrics Sensitizes by In-Situ Deposition of Copper and Silver Nanoparticles

**DOI:** 10.3390/nano11113097

**Published:** 2021-11-16

**Authors:** Azam Ali, Fiaz Hussain, Ambreen Kalsoom, Tauqeer Riaz, Muhammad Zaman Khan, Zakariya Zubair, Khubab Shaker, Jiri Militky, Muhammad Tayyab Noman, Munir Ashraf

**Affiliations:** 1 Department of Materials and Textile Engineering, Technical University of Liberec, 46015 Liberec, Czech Republic; zamankhan017@yahoo.com (M.Z.K.); jiri.militky@tul.cz (J.M.); 2 Functional Textile Research Group, Faculty of Engineering and Technology, National Textile University, Faisalabad 37610, Pakistan; fiazravian@gmail.com (F.H.); munir@ntu.edu.pk (M.A.); 3 Department of Physics, The Government Sadiq College Women University, Bahawalpur 63100, Pakistan; 4 Department of Chemistry, University of Gujrat, Gujirat 50700, Pakistan; touqeer.riaz@uog.edu.pk; 5 Department of Materials Engineering, National Textile University, Faisalabad 37610, Pakistan; zzubair@ntu.edu.pk (Z.Z.); khubab@ntu.edu.pk (K.S.); 6 Department of Machinery Construction, Technical University of Liberec, 46015 Liberec, Czech Republic; muhammad.tayyab.noman@tul.cz

**Keywords:** copper and silver nanoparticles, electroplating, electrically conductive fabrics, EMI shielding

## Abstract

In this study, we developed multifunctional and durable textile sensors. The fabrics were coated with metal in two steps. At first, pretreatment of fabric was performed, and then copper and silver particles were coated by the chemical reduction method. Hence, the absorbance/adherence of metal was confirmed by the deposition of particles on microfibers. The particles filled the micro spaces between the fibers and made the continuous network to facilitate the electrical conduction. Secondly, further electroplating of the metal was performed to make the compact layer on the particle- coated fabric. The fabrics were analyzed against electrical resistivity and electromagnetic shielding over the frequency range of 200 MHz to 1500 MHz. The presence of metal coating was confirmed from the surface microstructure of coated fabric samples examined by scanning electron microscopy, EDS, and XRD tests. For optimized plating parameters, the minimum surface resistivity of 67 Ω, EMI shielding of 66 dB and Ohmic heating of 118 °C at 10 V was observed. It was found that EMI SH was increased with an increase in the deposition rate of the metal. Furthermore, towards the end, the durability of conductive textiles was observed against severe washing. It was observed that even after severe washing there was an insignificant increase in electrical resistivity and good retention of the metal coating, as was also proven with SEM images.

## 1. Introduction

Metal-coated textiles are seeing an increased demand in technical and high-tech applications due to their novel properties of electrical conductivity and EMI shielding ability. The most potential applications are anti-static, UV radiation screen, hydrophobicity, radar reflectivity, flexible electrodes, antibacterial, and certain therapeutic applications like the development of electrodes for TENs (transcutaneous electrical nerve stimulation), ECG (Electrocardiography), and EMG (Electromyography) machines, etc. [1,2,3]. In general, the development of sustainable, conductive textiles is based on the selection of ecological and biodegradable resources. Synthetic textiles made by polymeric fibers create post-disposal problems. To overcome this issue, the researchers have started to utilize natural fiber sources to develop multifunctional electrically conductive textiles [4,5]. Textiles made up of cotton are well-known because of their use in biodegradable and sustainable products. Furthermore, virgin cotton fabric is non-conductive. Generally, insulators are converted to conductive materials by imparting the conductive ingredients on the surface [6]. Researchers have been focusing to make conductive textiles with silver and gold, and this has led to the production of commercial products. Some studies were conducted that used the combination of copper and silver to produce the pressure sensors [7,8]. In fact, these metals are much more expensive to be commercialized at industrial scales. From these perspectives, the use of copper is the best alternative to develop the economically conducive textile. Copper is highly conductive (close to silver), but is cheaper as compared to silver and gold. Copper is a highly conductive material and is currently used for electroplating over natural fibre based yarns and fabrics. The resulting fabrics behave as electrically conductive material with high electromagnetic shielding [9].

Mostly, metallization on textiles has been done by the use of conductive paints, conductive inks, metal lamination, spraying (the mixture of metal powder and binder), etc. These techniques are susceptible to the drawback of stiffness and poor air permeability. Sometimes yarns are directly spun from thin metal wires and blended into the textile structure, but it is difficult and uncomfortable to carry hard metal wires against the human body [10]. A variety of other methods include in situ deposition of metal particles, spraying (flame and arc spraying), sputtering, electrode vacuum deposition and electro-less plating. However, conductive textiles made in such traditional ways consist of defects such as high stiffness, oxidation of metallic surfaces, heavy, poor washing properties, poor rubbing properties, and weak air permeability, etc. [11]. Some of them have been applied for the sensitization of surfaces, firstly with vacuum deposition and sputtering, and secondly with applied silver plating on the surface of conductive material [12]. These techniques are better for achieving the surface conductivity, but fail to achieve the volume conductivity throughout the structure of the material. Among these techniques, electroplating over solution-sensitized particle-coated fabrics is considered to provide better results regarding coherent metal deposition, durability, even coating, volume resistivity, and is also applicable to complex-shaped materials. In a recent study, Ali Et al performed copper electroplating over copper particles coated with nylon fabric. They found the results of electrical resistivity (55 Ω/square) comparable to the present study (67 Ω/square) [13]. In this study, we are metallizing the cotton fabrics in a very simple and economical method. Generally, there is need to perform a number of pretreatment steps (pretreatment, sensitization, activation) before electroplating. These steps make the process lengthy, costly, and also not sufficient to develop durable and highly conductive textiles. In the current study we have eliminated the sensitization step by directly imparting silver particles and copper particles over the fabric surface. After the deposition of silver and copper particles, the fabric is ready for copper electroplating. This method of plating is focused on lower cost and environmentally friendly (palladium free, stannous free and formaldehyde free) copper plating over cotton fabric. Surface activation with nanoparticles is more stable and provides a better base for further electroplating. This is because nanoparticles cover more surface area and also penetrate properly into the fabric structure to provide volume conductivity. Furthermore, we are using copper and silver nano particles, which have a more positive redox potential than the copper which is going to deposit. Actually, if we do use any base particles which have a negative redox potential other than copper depositing, then the host element will be replaced by copper. The base will not give the homogeneous ground and will cause for the thickness variation for copper plating, and in turn electrical resistivity will be affected. Copper electroplating deposition on nano copper and silver particles is also better regarding comfort properties (drape, thickness and stiffness).

Secondly, a lot of work has been done for copper plating over stannous, cobalt and lead, which cannot be used for hygienic applications since tin (Sn) is hazardous and an irritant to the skin. In the existing method, we confirmed the antibacterial activity of developed fabrics. Fabrics can be used for environmental friendly applications like electrodes for (TENs) machines, and for military applications where we need electromagnetic shielding and hygienic west in case of injury of sliders. Furthermore, after electroplating with both methods, we have compared the functional and ageing properties of developed fabrics.

## 2. Experimental

### 2.1. Materials

Cotton fabric with plain weave structure (EPI × PPI = 28 × 23, warp and weft count = 23 s Ne, GSM = 150) were used. The fabric was purchased from Licolor, A, S, Liberec, Czech Republic. The chemicals used for metallization were of reagent grade.

### 2.2. Methods

#### 2.2.1. Pretreatment

Before the deposition of metal particles on cotton substrate, the fabrics are pre-treated with citric acid to enhance the functional groups over the cellulosic structure. At first, 15 g of citric acid (CH_3_COOH) was dissolved in 1 litter of water, and solution was stirred properly. The fabric was dipped in the solution at 100 °C for 120 min. Then the immersed fabric was rinsed with distilled water and dried at 80 °C for 40 min.

#### 2.2.2. Copper Particles Deposition

The copper particles were deposited by the chemical reduction method. Different concentrations of copper sulphate were dissolved in deionized water and fabric was dipped in solution for about 15 min and dried in the oven at 80 °C for 5 min. The procedure of dipping and drying was continuously carried out up to 15 cycles. Subsequently, the treated substrate was transferred to the 10 g/L sodium hydrosulfite solution. It is the beauty of sodium hydrosulfite reducing agent that it induces the most uniform and smallest particles among all reducing agents. The process of reduction was continued for about 40 min. This was termed as copper coated fabric activated for further copper electroplating.

#### 2.2.3. Silver Particles Deposition

The silver particles were also deposited by the chemical reduction method. Different concentrations of silver nitrate (AgNO_3_) were dissolved in deionized water. Then aqua ammonia (28 wt%) was added drop-wise in this solution and stirred continuously until a transparent solution of [Ag(NH_3_)_2_]^+^ was obtained. The fabric was dipped in solution for about 15 min and dried in the oven at 80 °C for 5 min. Hence the procedure of dipping and drying was continuously carried out up to 15 cycles. Subsequently, the treated substrate was transferred to the 15 g/L glucose solution. The process of reduction was continued for about 40 min. This was termed as silver particle-coated fabric activated for further copper electroplating.

#### 2.2.4. Electrolytic Copper Plating

**Figure 1:**
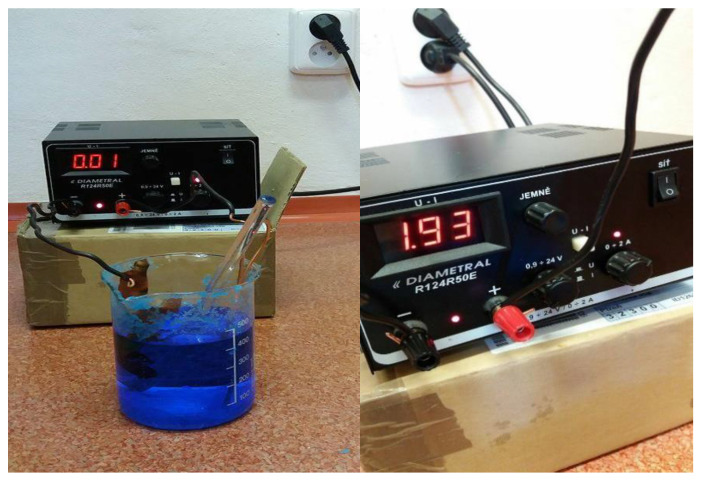
Electroplating process on conductive fabric.

The copper and silver particle-coated fabric was used as a substrate. Three different concentrations of Copper (II) Sulfate pentahydrate (CuSO_4_·5H_2_O) were dissolved in 1 L o\f distilled water. After this 10 g of 75% concentrated Sulphuric acid (H_2_SO_4_) was added very slowly. The electrolytic power source (having wires with copper clumps) was used. The power was rated at 24 V/2 Amp. A constant of 1.8 ± 0.2 A was maintained during the complete process. The cathode of source was connected with particles coated activated textile while anode was connected with copper metal bar. The solution of electroplating bath was stirred continuously. The whole setup is also shown in Figure 1.

Three different concentrations of copper sulfate: 10, 20 and 30 g/L, were dissolved in a fixed concentration of acid at 10 g/L. The time for electroplating was adjusted for 10, 20 and 30 min of the time interval. The experimental design for electroplated conductive textiles is given in Table 1.

### 2.3. Testing

#### 2.3.1. Surface Properties Evaluation

The infrared ray spectroscopy of citric acid pretreated cotton substrates were recorded by Nicolet Nexus 470 spectrometer equipped with an Attenuated Total Reflection Pike-Miracle accessory (PIKE Instrument, Illerkirchberg, Germany). The particle size of copper and silver particles (prior to further copper electroplating) was measured by the dynamic light scattering (DLS) technique. The particles were rubbed from the surface of fabric and dilute dispersion was prepared in deionized water. The solution was ultrasonicated for 20 min with a bandelin ultrasonic probe before testing. The surface morphology of metal coated fabrics was observed by scanning electron microscopy (SEM) (TESCAN, Dortmund, Germany). An accelerated voltage was applied by Tescan VEGA III SEM apparatus. EDX analysis was performed to measure the elemental percentage by weight. XRD analysis was performed in the range of 10–80 degrees by adjusting the step size of 0.02. The diffractometer equipped with copper radiation at a rated power of 40 kV/30 mA was used.

#### 2.3.2. Electrical Properties Evaluation

**Figure 2:**
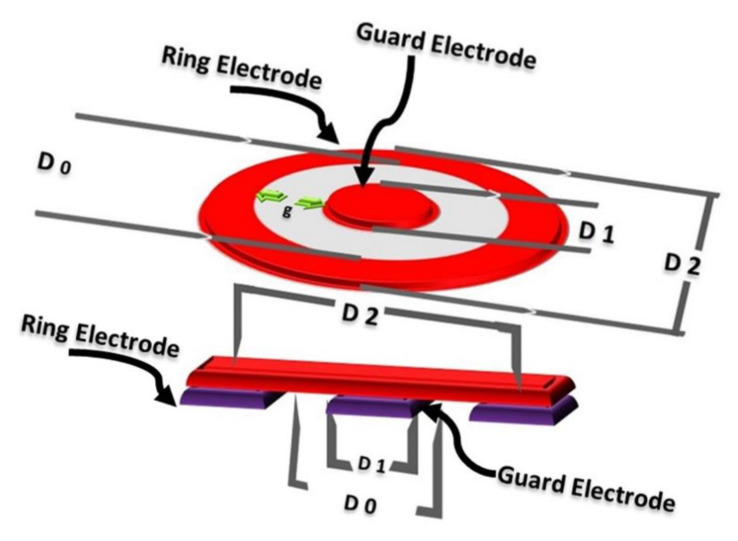
Setup for electrical conductivity test.

Electrical resistivity was measured for all the conductive fabric samples (samples coated with copper and silver particles and copper electroplated fabrics). The resistivity was measured by concentric electrode method by ASTM D257-07. The fabric samples were kept under constant pressure of 2.3 kPa at 20 °C with relative humidity *RH* = 60 ± 5%. The apparatus had two concentric circular electrodes and sample was placed between these electrodes. Each sample was tested five times from different places and final measurement was the average of these measurements. The DC power source rated with constant voltages of 100 V was applied. The voltage potential followed by change in current with resistance was measured, while the resistivity *ρ_s_* [Ω] was measured according to following equation.

(1) $$\rho_{s}=R_{s\;}\left(\frac{\pi D_{0}}{g}\right)$$

where *R_S_* is the resistance in ohm [Ω], *D*0 is the central distance calculated as (*D*2−*D*1)/2, *D*1 is the outer diameter of the center electrode [mm], *D*2 is the inner diameter of the upper ring electrode [mm], *g* is the adjusted distance between *D*1 and *D*2 [mm]. The Schematic of instrument used to check the electrical resistivity is shown in Figure 2.

#### 2.3.3. Weight Gain Percentage

The percentage of weight gain of electroplated conductive textile was examined according to the following equations:

(2) $$\rho_{s}=R_{s\;}\left(\frac{\pi D_{0}}{g}\right)$$

(3) $$w=\frac{m-m_{0}\;}{m_{0}}\times 100\;$$

where *m* represents the final mass, *m*_0_ is the original mass of conductive textile and *w* denotes the percentage of total weight gain.

#### 2.3.4. Measurement of EMI Shielding

Electromagnetic shielding interference was measured according to ASTM D4935-10. All conductive textiles were subjected to a frequency range of 30 MHz to 1500 MHz by using coaxial transmission line method. The apparatus was generating the electromagnetic signals through a network analyzer. The shielding interference was analyzed by calculating the ratios between the incident (*i*) and transmitted (*t*) rays. Equation number (4) represents the ratios of power densities to measure the shielding ability (*SE*).

(4) $$SE\;\left(dB\right)=10\;log\frac{P_{t}}{P_{i}}$$

where *P_t_* and *P_i_* are power densities (W·m^−2^) measured in the presence of sample (transmitted), and without the sample (incident) respectively.

#### 2.3.5. Heat Generating Performance of Conductive Textiles

The heat generating performance was observed by a FLIR thermo camera (W-Technika, Prague, Czech Republic). The theory of resistive heating is based on the voltage difference. Applying the voltage difference across the ends of the fabric causes resistive heating. The change in temperature across the surface of the fabric was observed. Hence we applied different voltages at different intervals of times and the amount of heat generated is related to I^2^ as given in Equation (5).

P = I^2^ R (5)

where P is the total power dissipation, I is the current passing through conductive fabrics and R denotes the total resistance of the operating heater.

#### 2.3.6. Durability of Conductive Fabrics

The durability of conductive fabrics was observed against severe washing (according to ISO 105-C01). The substrates were given standard washing according to ISO 105-C01 to check their stability in service. Standard detergent was used with a liquor ratio of 40:1. The temperature of the bath was kept constant at 45 °C with a speed of 800 rpm for 30 min. Subsequently, all samples were rinsed with distilled water, dried in the oven at 70 °C for 20 min. Furthermore, the durability was confirmed with electrical conductivity and SEM images.

## 3. Results and Discussion

### 3.1. Electrical Conductivity of Copper-and Silver Particles Coated Fabrics

**Figure 3:**
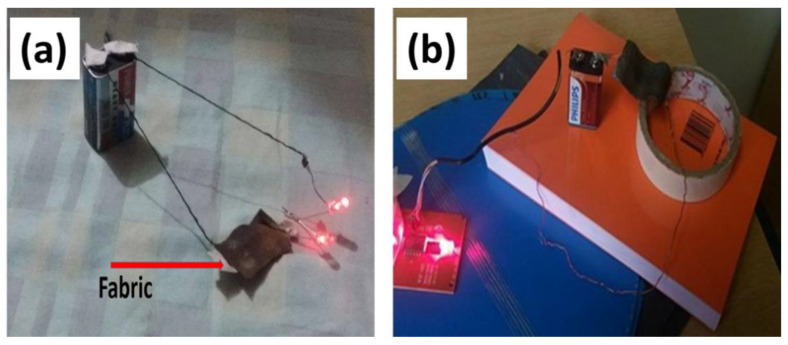
Flow of electricity through (**a**) silver particles (**b**) copper particle-coated fabrics.

The electrical conductivity of copper and silver nanoparticles coated cotton fabrics was checked through a direct current source. The fabric was connected through copper wires with a constant battery source. The glowing of bulb verifying the flow of electric current through the fabric. The electrical conductivity through fabric can be observed was as shown in Figure 3.

The untreated cotton fabric is non-conductive and behaves like electrically insulating material [14]. However, the insulators become conductive once they are coated with conductive materials. Therefore, the copper and silver particle-coated insulating fabrics were supposed to be conductive. The electrical conductivity was observed for all samples (samples made from the coating of Ag-NPs, samples made from the coating of Cu-NPs, and samples made after electroplating of copper over silver and copper nanoparticles coated fabrics). The measurements included surface resistivity, volume resistivity, comparison of resistivity values between surface resistivity *ρ_s_* and volume resistivity *ρ_v_*, in addition to a measurement of volume resistivity and surface resistivity with several bending cycles. At first, surface resistivity and volume resistivity of silver and copper coated fabrics was measured, which were the mean values of three measurements. The result showed the least surface resistivity at 17 g/L of silver salt and 10 g/L of copper salt as compared to higher concentrations. It means that at less concentration of salt we were able to achieve a good network of metal particles to conduct an electrical current. At higher concentrations of salts there is more nucleation of ions inside the solution. These ions tend to decrease the total surface energy and eventually agglomerated. Hence the formation of big particles over the fabric surface and the resulting non-homogenous coverage. Secondly, by further increasing in the number of dipping cycles, the heavy and non-homogeneous loosely held particles will erode back into the solution. This will cause uneven coating and fail to reach the conductive threshold. In fact, a higher concentration of salt tends to form the larger-sized particles. Surprisingly, at lower concentrations, as with 17 g/L of silver nitrate (AgNO_3_) and 10 g/L of copper sulfate (CuSO_4_), the salt produced more conductive fabrics. At a lower concentration the salts can produce the percolated network by the creation of continuous connectivity between the small-sized particles.

**Figure 4:**
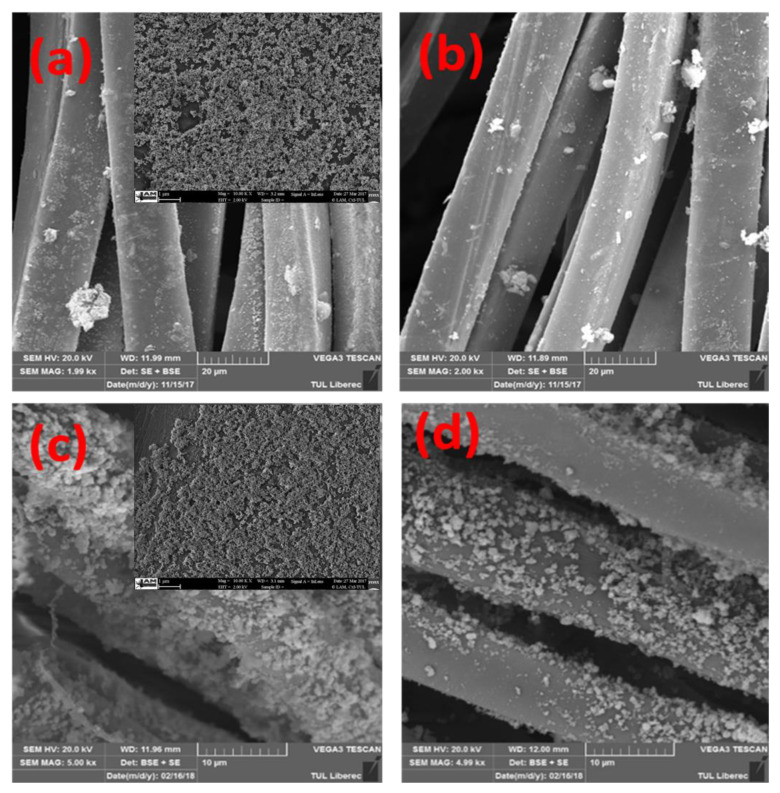
SEM images of silver coated fabrics for (**a**) 17 g/L (**b**) 34 g/L silver nitrate concentrations and copper coated fabrics for (**c**) 10 g/L (**d**) 20 g/L copper sulfate concentrations.

**Figure 5:**
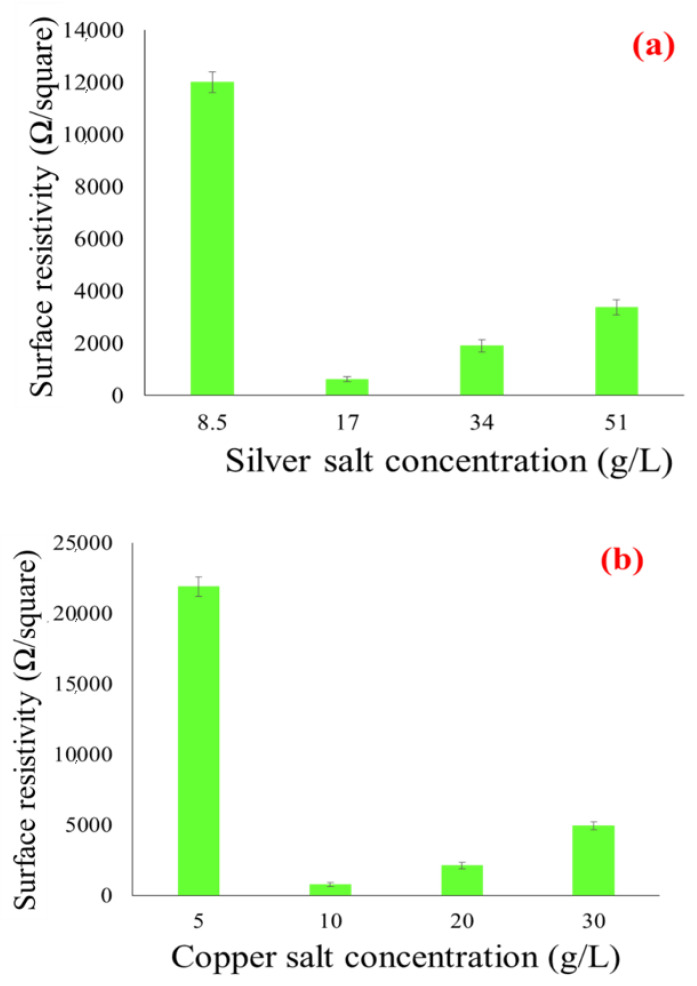
Surface resistivity of silver coated fabrics for different (**a**) silver nitrate concentrations and copper coated fabrics for different (**b**) copper sulfate concentrations.

This can be further justified from SEM images shown in Figure 4a–d, where the formation of a more percolated network of smaller particles can be found in the case of 17 g/L of silver salt than 34 g/L silver nitrate and 10 g/L of copper salt than 20 g/L copper sulfate. However, the experiments were also performed at 8.5 g/L of silver nitrate and 5 g/L of copper sulfate to check the effect of further lower concentrations of silver nitrate and copper sulfate on electrical resistivity. Amazingly, the results were quite different. At 8.5 g/L of silver nitrate and 5 g/L of copper sulfate, the resistivity was too high (12,000 Ω and 21,900 Ω, as shown in Figure 5a,b). This means 17 g/L for silver nitrate and 10 g/L of copper sulfate are the most suitable concentrations for the formation of percolated network by the creation of continuous connectivity between the small-sized silver and copper particles.

**Figure 6:**
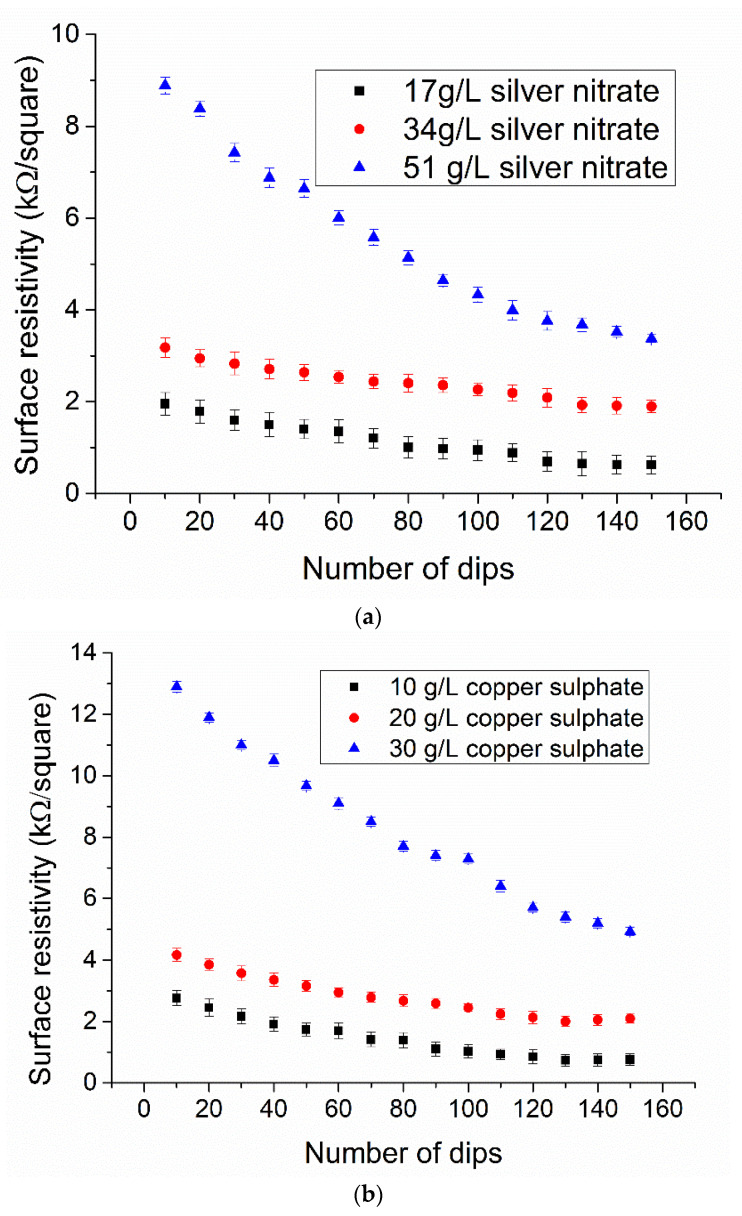
Effect of (**a**) silver nitrate, (**b**) copper sulphate concentrations and dipping on electrical resistivity (bars are limits of 95% confidence interval).

Furthermore, the effect of the number of dipping cycles against electrical resistivity was also observed, where the resistivity was found to reduce with an increase in the number of dips for all concentrations of silver and copper salt solution. This kind of behavior specified the fact of more dense and uniform deposition of nanoparticles at a higher number of dips, as shown in Figure 6a,b. As the lower concentration of salt showed better results for electrical conductivity, in further sections a detailed discussion is undertaken considering samples coated with 10 g/L of copper sulfate and 17 g/L of silver nitrate.

**Figure 7:**
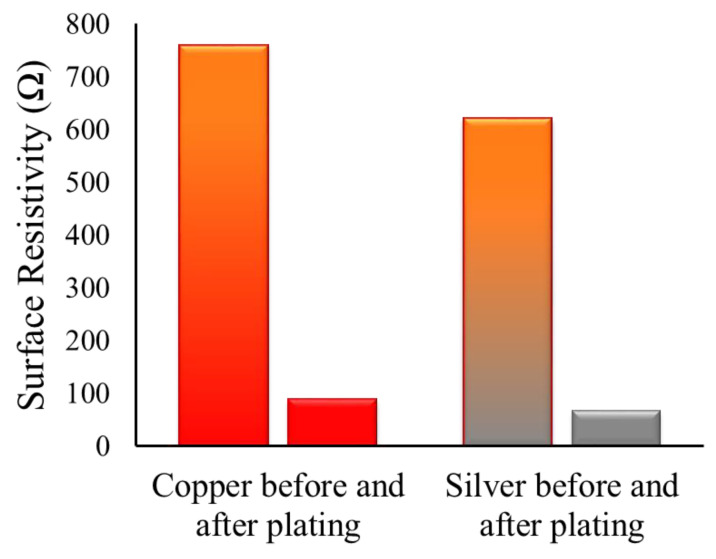
Reduction in electrical resistivity before and after electroplating.

In this study, the electrical properties of conductive fabrics were enhanced by depositing a fine film of silver and copper nanoparticles over the fabric surface, followed by the successive electroplating of copper metal (Figure 7). As it is essential, the substrate should be electrically conductive and act as a catalyst to deposit further metal on it. The electroplating of copper (Cu) provides a compact layer of metal on the surface of the substrate. The impact of further metal deposition (by the process of electroplating) on electrical resistivity values is now discussed. The concentration of acid and the time both are the major factors influencing copper electroplating. During the process of electrolysis, water breaks into the hydrogen (H_2_) and hydroxyl (2 OH^−^) ions. The hydrogen leaves the solution while the remaining OH^−^ ions raise the pH of the solution. Therefore, hydroxyl ions (OH^−^) must be balanced by adding acid. Furthermore, acid is also necessary because of its inherent property to provide the conductivity during the process of electroplating. It is clear from Table 2 that the higher concentration of copper sulfate 30 g/L at 5, 10 and 15 min time intervals resulted in the lowest electrical resistivity.

### 3.2. Weight Gain Percentage

**Figure 8:**
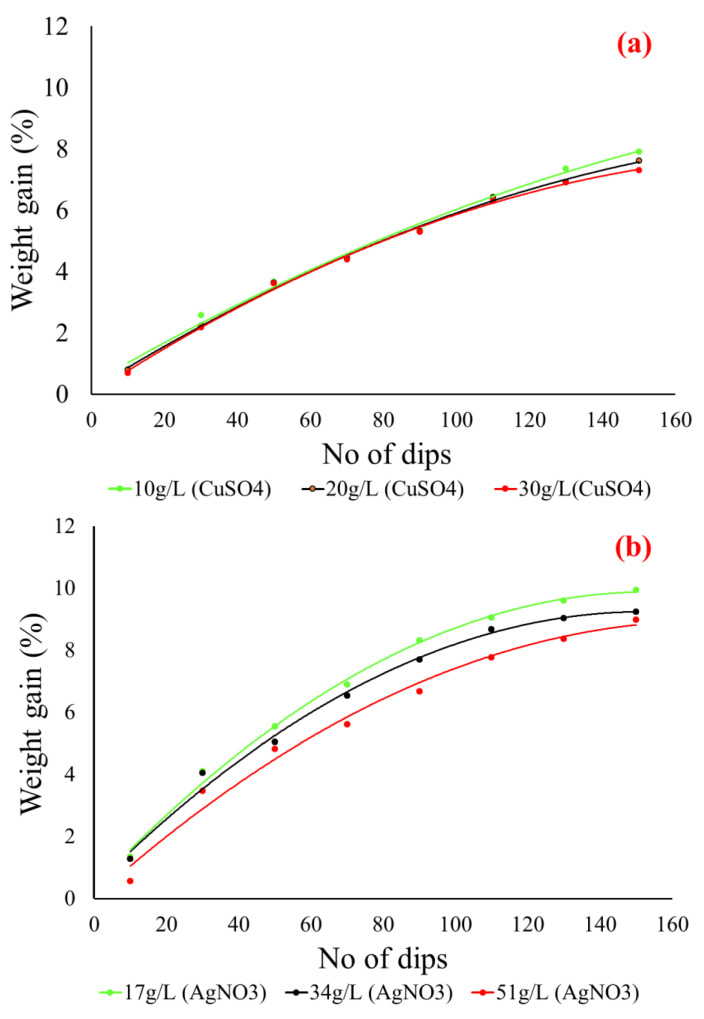
The weight gain percentage with increase in number of dipping cycles (**a**) Cu-NPs coated cotton fabric, (**b**) Ag-NPs coated cotton fabric.

The weight gain percentage of the Cu-NPs coated cotton fabric, the Ag-NPs coated cotton fabric and the electroplated cotton fabric was investigated. At first, the weight gain percentage was measured for all Ag-NPs coated fabric and Cu-NPs coated fabric samples. The effect of the number of dipping cycles against weight gain percentage was measured for each concentration of copper sulfate (10, 20 and 30 g/L) and silver nitrate (17, 34 and 51 g/L). It is clear from Figure 8 that the weight of the cotton fabric was increased in both cases with an increase in dipping cycles in respective salt solutions (copper sulfate and silver nitrate). It was observed that the weight gain percentage against each concentration of copper sulfate (10, 20 and 30 g/L) and silver nitrate (17, 34 and 51 g/L) was almost the same.

The maximum weight gain percentage for copper particles coating with 10, 20 and 30 g/L copper sulfate was 7.9%, 7.5%, and 7.3%, respectively. While the maximum weight gain percentage for silver particles coated with 17, 34 and 51g/L silver nitrate was 9.9%, 9.2%, and 8.9%, respectively. At lower concentrations of each salt copper sulfate and silver nitrate, the weight gain percentage was slightly higher as compared to higher concentrations. Overall, it was observed that weight gain percentage against each concentration of copper sulfate (10, 20 and 30 g/L) and silver nitrate (17, 34 and 51 g/L) was almost the same. In contrast to this, there was a huge difference between the electrical resistivity results. At lower concentrations of each salt, 10 g/L copper sulfate and 17 g/L silver nitrate fabric samples showed lowest electrical resistivity. The reason is that at higher concentrations of salts there is more nucleation of ions inside the solution. These ions tend to decrease the total surface energy and eventually agglomerate. Hence the formation of big particles over the fabric surface, and this causes non-homogenous coverage. Secondly, by a further increase in the number of dipping cycles, the heavy and non-homogeneous loosely held particles will erode back into the solution. This will cause uneven coating and continue fail to reach the conductive threshold. This behavior can be attributed to the formation of large copper particles at higher concentrations of the copper sulfate solution.

**Figure 9:**
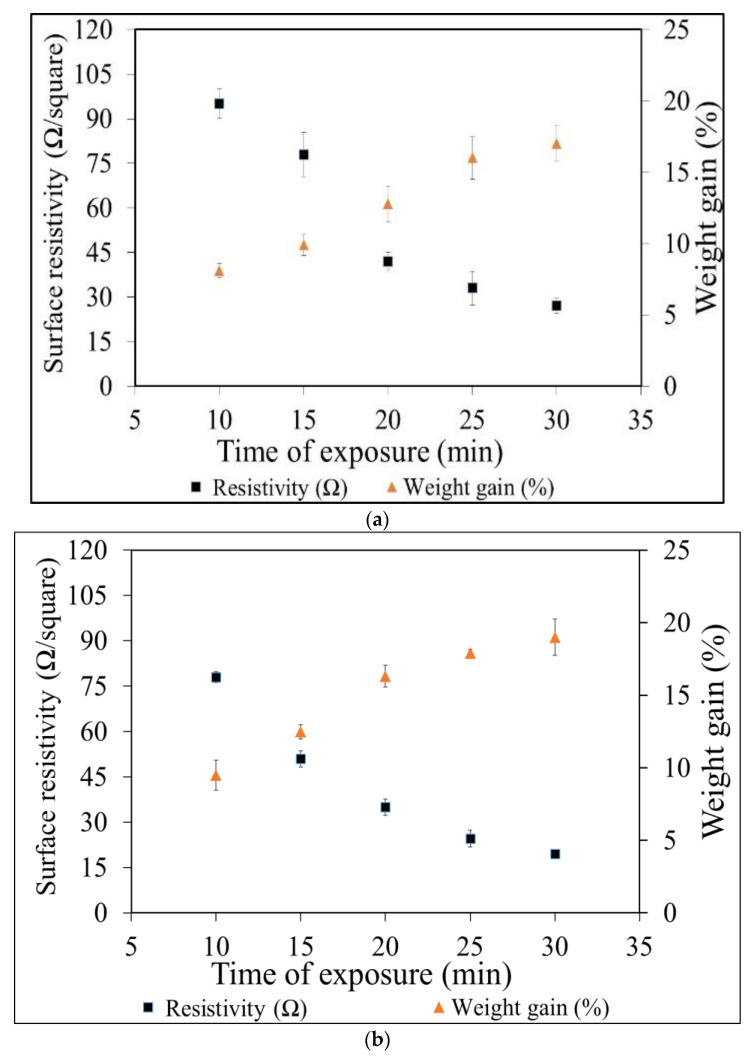
Mass gain percentage with time of copper plating over (**a**) Cu-NPs coated woven cotton fabric, (**b**) Ag-NPs coated woven cotton fabric (bars are limits of 95% confidence interval).

The percentage of fabric weight gain was also measured with copper plating. The fabric weight gain was measured for copper plating over the silver coated fabric and copper plating over copper coated fabric, and their respective graphs are shown in Figure 9. The electrical resistivities were decreasing with increasing time of electroless plating for all the cases. The lowest electrical resistivity was achieved with a maximum value of weight gain percentage at 30 min of electroplating. From the trend lines it is clear that as we are increasing the time of electroless plating, the percentage mass gain is going to increase and the electrical resistivity is going to decrease. The mass gain percentage of copper plating over silver coated fabric is higher than mass gain percentage for copper plating over copper particle-coated fabric.

### 3.3. Surface Morphology

#### 3.3.1. FTIR Measurements of Cotton Fabrics

**Figure 10:**
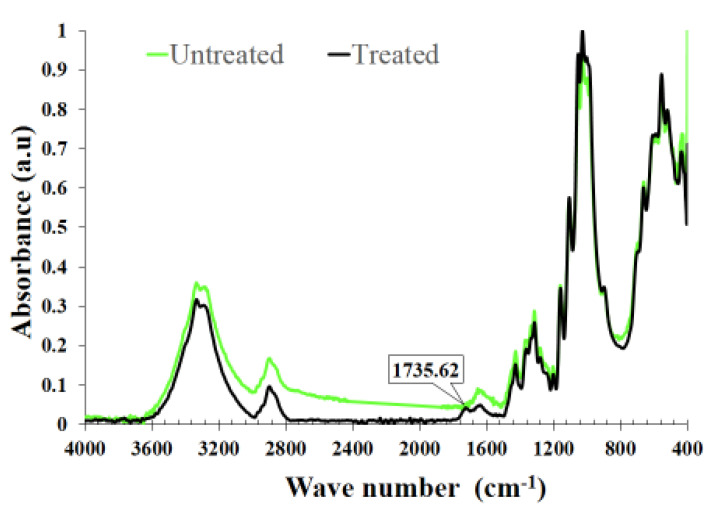
FT-IR spectra of treated and untreated cotton fabric.

The FTIR spectra of the cotton fibers treated with citric acid and of the control cotton fibers are shown in Figure 10. The comparison of infrared ray spectrum showed a broad peak centered at 3300 cm K1 corresponding to O–H stretching. Also, we detected another broad peak at 3000–2800 cm^−1^. This band is attributed to the region of C–H stretching. The vibrations located around the peak at 1640 cm^−1^ are due to the adsorbed water molecules. It is due to the C=C stretching that point out the presence of aromatic rings. An additional band appears at 1732 cm^−1^ for the citric acid-grafted cotton this points out the absorption of the carboxylic group [15].

#### 3.3.2. SEM Structures

Scanning electron microscopy was performed before and after electroplating to measure the morphological changes over the surface of the textile substrate. The SEM analysis of copper and silver particle-coated cotton textiles are already shown in Figure 4. The SEM images depict the nanometer scale of silver and copper nanoparticle deposition. The deposition is uniform and homogeneously covered all the surface areas of the fibers. Furthermore, the dense deposition will provide a better platform for the further electroplating of copper to form the percolated network of conductive fabric. In the second stage, SEM analysis was further carried out for copper-plated fabrics.

**Figure 11:**
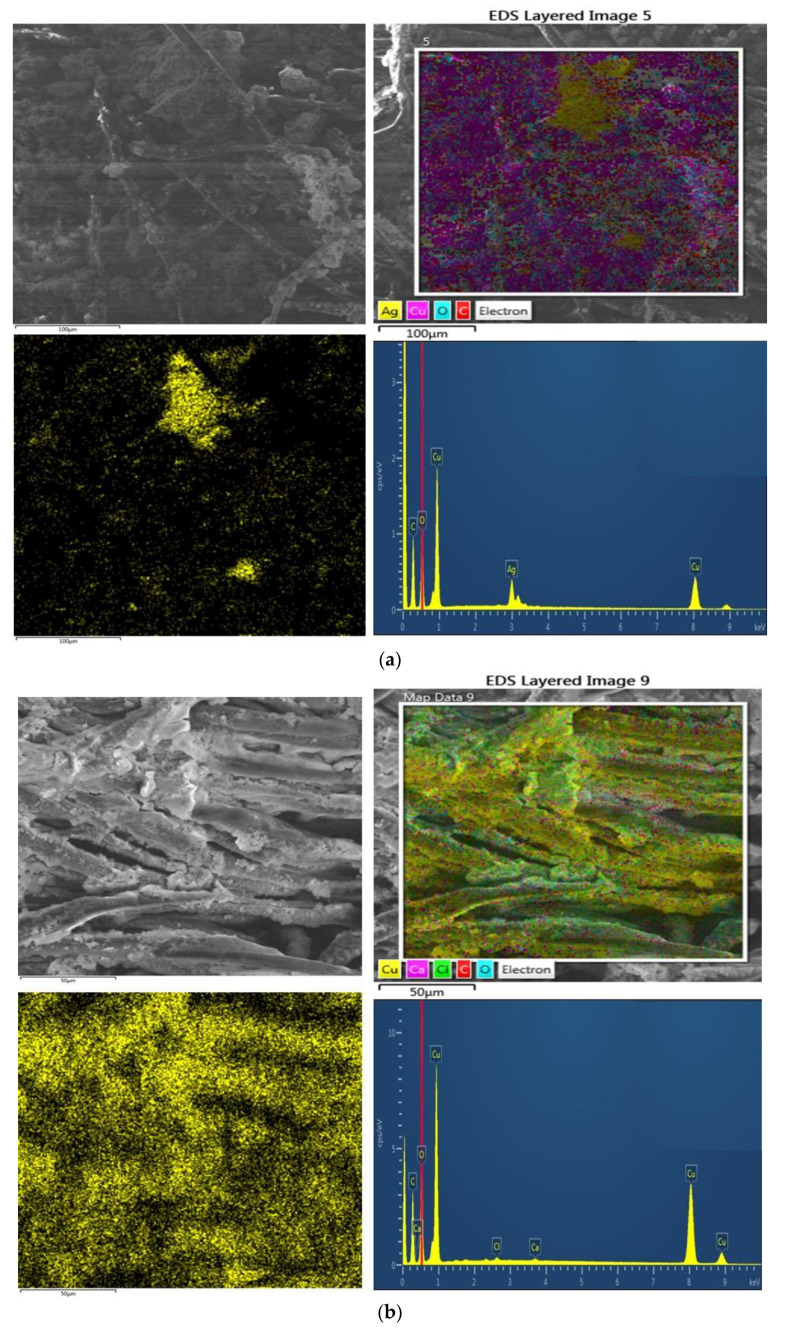
SEM image of (**a**) copper electro-plating over the silver particle-coated fabric (A9) and (**b**) copper electro-plating over copper particle-coated fabric (C9).

Which are showing the copper electro-plating over the copper particles coated fabric (C9) and copper electro-plating over silver particles coated fabric (A9). Figure 11a,b is showing the more dense deposition of copper as compared to previously particle-coated fabrics. The reason is that samples were already coated with a homogeneous layer of copper and silver nanoparticles, which covered more surface area. They provided a wide platform for the deposition of additional copper. Moreover, with SEM analysis, the elemental composition of all plated fabrics was also determined by EDX analysis. The copper peaks are visible in each spectrum. The content of copper is more in copper plating over silver and copper plating over copper-coated fabric as compared to previously only particle-coated fabrics.

#### 3.3.3. XRD Analysis

**Figure 12:**
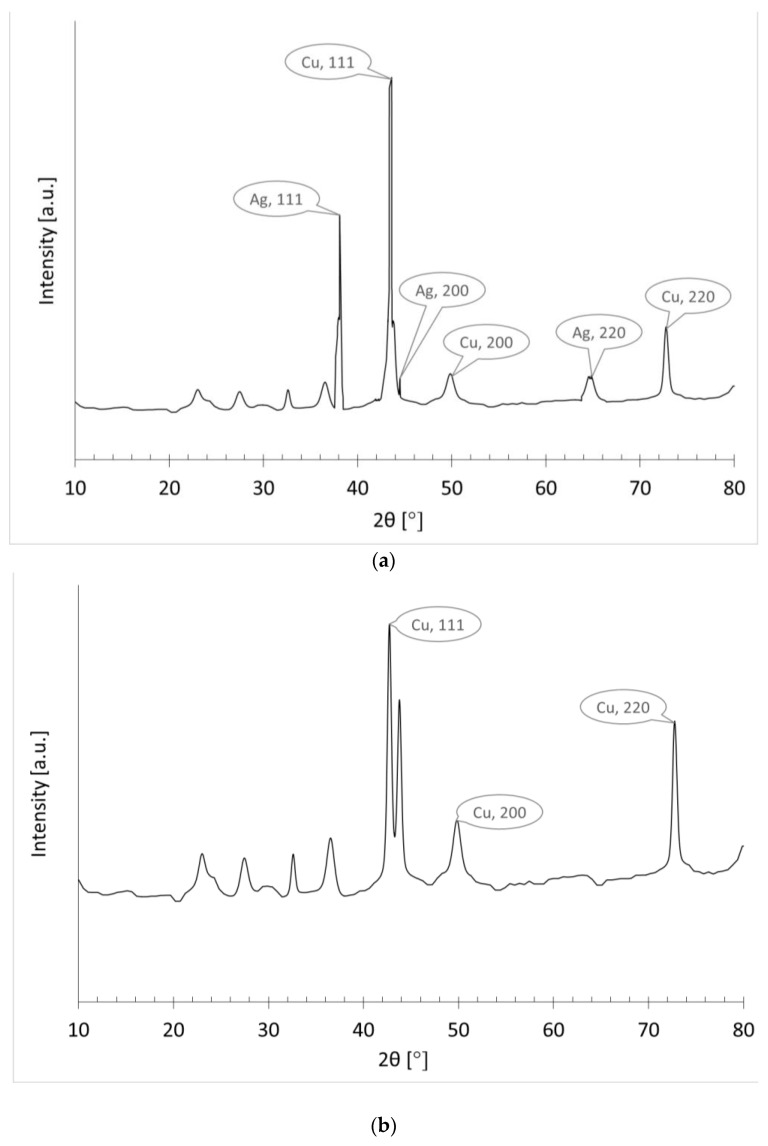
XRD pattern of (**a**) copper electro-plating over the silver particles coated fabric (A9) and (**b**) copper electro-plating over copper particles coated fabric (C9).

The phase purity and composition of deposited copper was analyzed by the XRD pattern of copper electroplated fabrics. Figure 12a,b shows the XRD pattern of copper electroplating over silver particle-treated fabric and copper particle-coated fabrics. The wide angle XRD analysis was performed in the range of 20 degrees to 80 degrees (by using 2θ with a step of 0.02 degrees). The phase composition of the deposited copper over silver nanoparticle-coated fabric can be seen from the perfect indexing of all the diffraction peaks to their structures. The functional fabric (copper plated over silver particles) indicated four new peaks at 2θ values of 38.1°, 44.3°, 64.5°, and 77.5° for silver. These diffraction peaks are related to the peaks obtained from the planes (1 1 1), (2 0 0), (2 2 0) and (3 1 1) for cubic structure of silver [16]. In the same sample, we also found all diffraction peaks to the copper structure. The diffraction peaks observed at 2θ of 43.3°, 50.5°, and 74.2° respectively denotes the (1 1 1), (2 0 0) and (2 2 0) planes of copper [17]. The sharpness of the peaks is representing the crystalline nature of copper particles. Intrinsically, no characteristic peaks of other impurities were detected, except the peak of cuprous oxide Cu_2_O (appeared at 38°). Moreover, Figure 12b is showing only diffraction peaks related to the copper structure. The diffraction peaks appeared at 2θ of 43.3°, 50.5°, and 74.2° represented (111), (200) and (220) planes of copper, respectively [18], because this sample was made by electro-less copper plating over copper deposited fabrics.

### 3.4. Mechanism and Reactions Involve for Metallization

#### 3.4.1. Mechanism for the Attachment of Silver Particles

**Figure 13:**
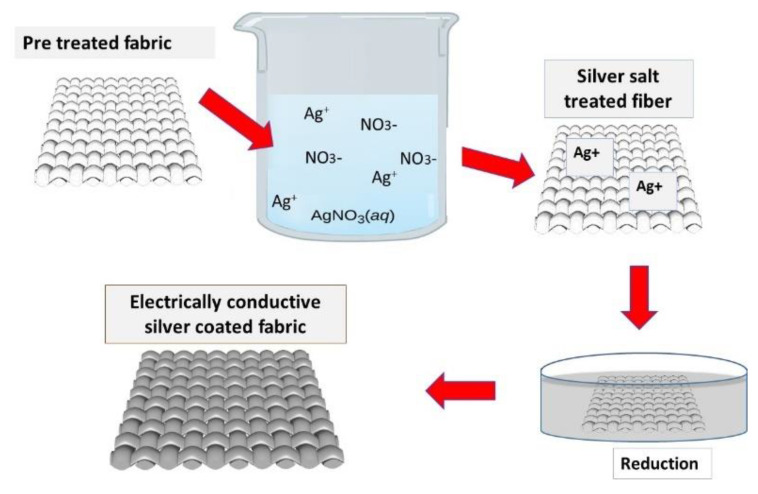
The attachment of silver particles on cotton fabric.

The attachment of silver particles on cotton fabric is explained in Figure 13. The solution of AgNO_3_ gives the Ag^+^ and NO_3_^−^ ions. The pretreated cotton fiber will absorb the silver ions to some extent based on the heterogeneity of the cellulose phases in the fabric. Furthermore, during the formation of equilibrium reaction, the ammonia also forms the complex ion [Ag(NH_3_)_2_]^+^ with Ag^+^. As a result, the Ag^+^ ion will also act as the oxidizing agent to form Ag°. Cotton, which is already rich regarding anionic sites due to pretreatment, will provide better space to continue the uptake of silver ions and the complex ion [Ag(NH_3_)_2_]^+^ [19]. The ions will be attached by van der Waal forces and due to ionic bond between the Ag^+^ and negative groups available on the cotton surface. Moreover, the reducing agents will further reduce the silver and its complex ions to silver metal.

#### 3.4.2. A Mechanism for the Attachment of Copper Particles

**Figure 14:**
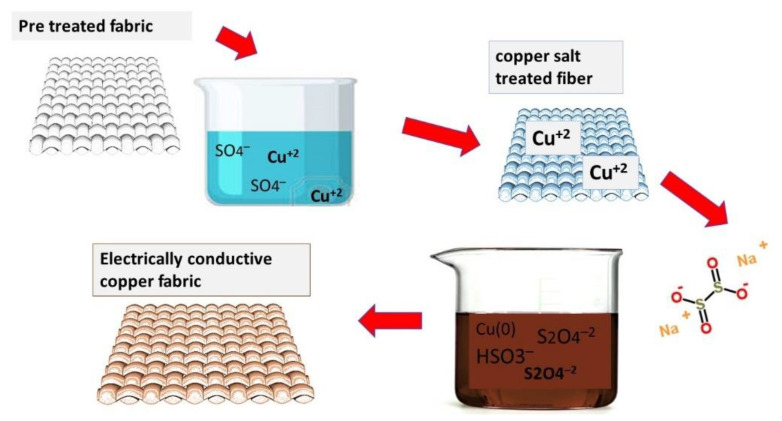
Mechanism for the attachment of copper particles on cotton fabric.

The attachment of copper particles on cotton fabric is explained in Figure 14. The solution of CuSO_4_ gives Cu^+2^ and SO4^−2^ ions. The cotton substrate which is already rich regarding anionic sites due to pretreatment will provide better space to continue the uptake of copper ions [20]. Subsequently, the copper ions (Cu^+2^) tend towards the surface of cotton fibers. The cellulosic structure of fibers captivate the copper ions based on the heterogeneity of the cellulose phases [21]. Similarly, sodium dithionite Na_2_S_2_O_4_ (a reducing agent) dissolves in water and breaks into suitable reducing ions (dithionite S_2_O_4_^−2^ and Na^+^). Later, when fabric containing the Cu^+2^ ions (on the surface) was transferred into the aqueous solution of sodium dithionite Na_2_S_2_O_4_, the redox reaction occurred between the oxidizing Cu^+2^ ions and reducing S_2_O_4_^−2^ ions. The reducing ions ultimately reduce the Cu^2+^ ions to Cu^+^ and then to copper metal [22].

#### 3.4.3. Copper Electroplating on Conductive Fabrics

**Figure 15:**
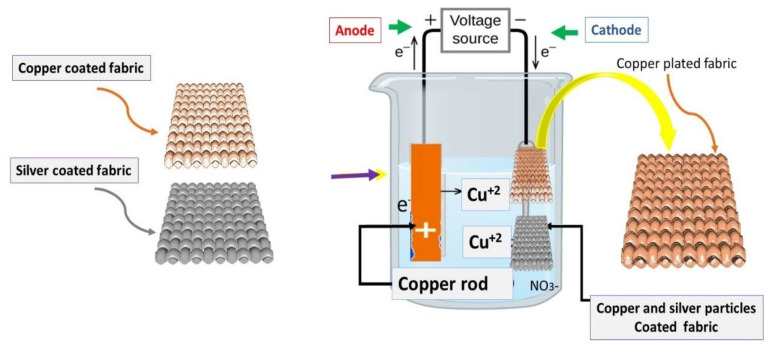
Schematic showing the electroplating of the copper by using copper electrode at cathode and conductive fabrics at anode.

The copper (II) sulfate pentahydrate (CuSO_4_·5H_2_O) dissociates into water contents (H_2_O) and possibly to a further homogeneous mixture of individual ions Cu^+^ ions and sulfate SO_4_^−^ ions or molecules. Hence, an active electrolyte is formed in this process of salvation. During the electrolysis process, water electrolysis occurs in H_2_ and 2 OH^−^ ions. The hydrogen leaves the solution in gaseous form, while remaining OH^−^ ions will raise the pH of the solution. So, OH^−^ must be balanced by adding a suitable acid. Secondly, the acid is also necessary due to its inherent property to support the electrical conductivity during the process of electroplating. The copper bar (acting as an electrode) was attached to the (+) anode, while the electrically conductive fabric (made by deposition of copper and silver nanoparticles) was attached to the (−) cathode. Cu^+2^ ions were discharged from the anode (copper bar) and deposited on the cathode (conductive fabric). During the process of electrolysis, the cathode had been thick (from receiving the copper ions), while the anode was dissolved slowly (by losing the copper ions). The process involved in electrochemistry is shown below in Figure 15.

### 3.5. Heating Performance

**Figure 16:**
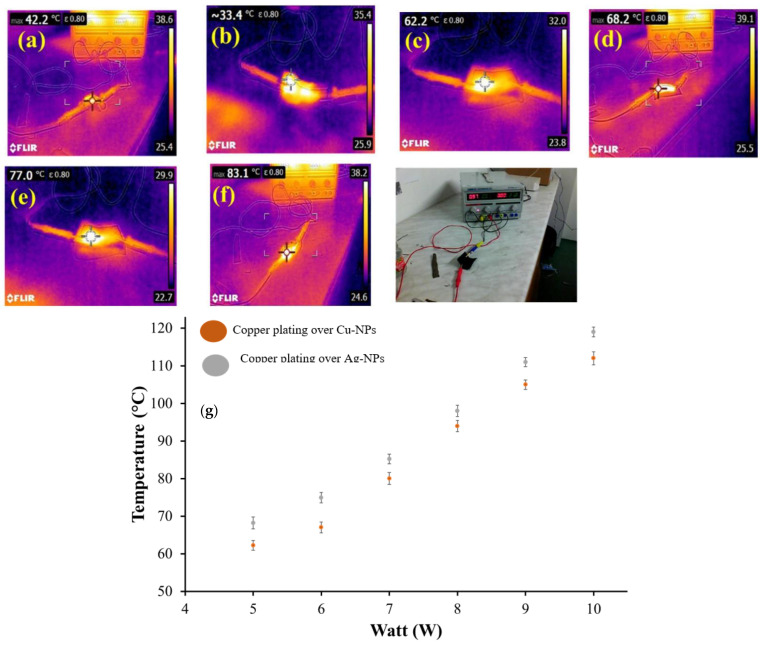
Surface temperature of copper plated fabrics samples: step one at 5 V and 1 min for (**a**) Cu-NPs coated fabric, (**b**) Ag-NPs coated fabric (**c**) electro-plated copper for Cu-NPs coated woven cotton fabric, (**d**) electro-plated copper for Ag-NPs coated woven cotton fabric respectively, step two at 5 volt and 10 min (**e**), (**f**) and step three at 5–10 watt (**g**).

The heating performances of the Cu-NPs coated cotton fabric, Ag-NPs coated cotton fabric and the electroplated cotton fabric was investigated. Joule heating of conductive fabrics was measured by applying the voltage at the ends of the fabric. The variation in surface temperature of fabrics was recorded (see Figure 16). Firstly, we applied fix voltage of 5 V with 0.9 to 1 A of current for 1 min at the ends of each fabric and the recorded increase in temperature was studied. The maximum temperature observed for Ag-NPs (17 g/L) coated fabric and Cu-NPs (10 g/L) coated fabric was about 42.2 °C and 33.4 °C respectively (see Figure 16a,b), while the temperature for copper plating over Ag-NPs coated fabric and copper plating over Cu-NPs coated fabric was recorded at about 62.2 °C and 68.2 °C see Figure 16c,d). In the second step, the temperature was measured as a function of time up to 10 min with a constant applied voltage of 5 V with 0.9 to 1 A of current. The maximum temperature (83 °C for copper plating over Ag-NPs coated fabric and 77 °C for copper plating over Cu-NPs coated fabric) were obtained when the applied voltage was 5 V for 10 min, as shown in Figure 16e,f). The temperature on the fabric surface increased up to 83 °C and 77 °C within 1 min, then it slowly increased. For study the heat-generating capacity and stability against resistive heating, the conductive fabrics were subjected to the electric source (able to provide constant current and voltages). In this way, the temperature was recorded as a function of voltages (with varying voltages from 5 to 10 V) and current (was maintained at 1 A). Hence the outcome (of different voltages DC input varied from 5–10 V and at constant current 1 A) was calculated in watt for plated fabric samples (copper plating over Ag-NPs coated fabric and copper plating over Cu-NPs coated fabric) and the steady state surface temperature was noted Figure 16g. The experiment was carried out up to 10 watts. The maximum surface temperature of 118 °C was observed for copper plating over Ag-NPs coated fabric and 111 °C for copper plating over Cu-NPs coated fabric. In a similar study, cotton fabric was functionalized with carbon nanotubes (CNT), and it completely loses the heating power of the conductive fabric after 220 s. In contrast, in the present study, the stability of the conductive fabric is retained even after 60 min. In another study, Ali and co-workers tested the voltage effect on their functionalized conductive fabric made from silver- and copper-coated particles and reached a maximum surface temperature of 112 °C with 80 mm terminal separation and at 10 volts [23]. The applied electric potential difference caused the acceleration of the charge carriers in conductive textiles. Therefore, heat is produced by the inelastic collision of charge carriers with phonons and defects present on the conductive textiles. During the increase in voltage the number of charge carriers also increased, which in turn increased the surface temperature [24].

### 3.6. Electromagnetic Shielding of Conductive Fabrics

**Figure 17:**
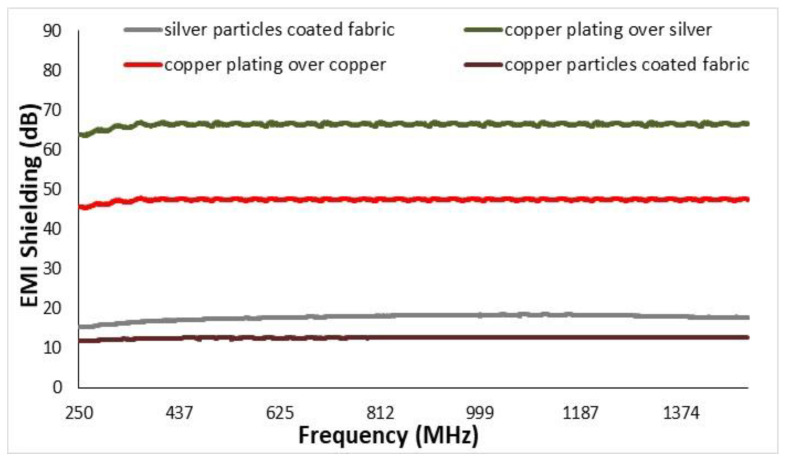
EMI SH Values for Cu-NPs coated fabric and Cu-Electroplated fabric samples.

Before electroplating, the EMI shielding values of copper and silver particles coated fabric were about 12.65 dB and 18 dB, respectively. After performing the electroplating of copper, the same fabrics showed higher values of EMI shielding. The EMI shielding for fabrics of copper-plated over silver and copper-plated over copper fabric was noticed to be about 66 dB and 49 dB, respectively. This behavior was attributed to their higher electrical conductivity values, and therefore increased reflection of electromagnetic waves. The frequency range of shielding effectiveness was adjusted up to 1500 MHz, as shown in Figure 17. The higher electromagnetic shielding is always coupled with high electrical conductivity. The results showed that with a decrease in resistivity (that is an increase in electrical conductivity) a considerable change in EMI SE occurs. There is a significant difference between the EMI SE values of the without copper plating and with copper plating samples.

### 3.7. Ageing

**Figure 18:**
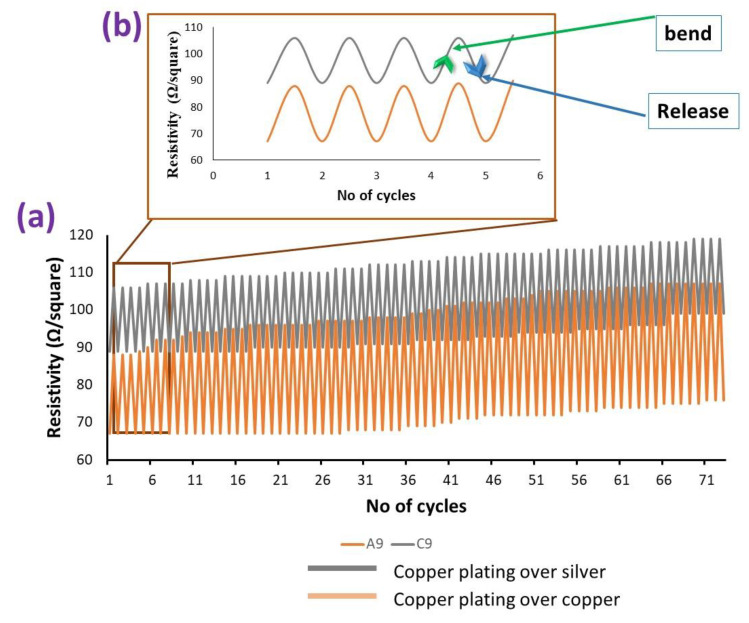
(**a**) Stability in resistivity during mechanical action of different no of cycles (bend and release), (**b**) blue and green arrows are representing the bend and release modes (with slightly shifted wavelengths to avoid from overlapping and for better understanding).

Various bending cycles were subjected to conductive fabrics to check their durability. During the start of first cycle (of wavelength), initial surface resistivity of the samples C9 (copper plating over copper particles coated woven fabric) and A9 (copper plating over silver particles coated woven fabric), were 67 and 88 Ω/squared, respectively. During the second step, we completely bend the samples to check their performance against severe machinal action and measured their corresponding resistivity, which were noted as 85 and 102 Ω/squared. Then the samples were released and they reverted to the original position (ie., straight) and at this position resistivity was measured as 67 Ω/squared and 88.1 Ω/squared, respectively. This was determined to be one complete cycle. So, during one complete cycle, we measured the surface resistivity of each sample at three different positions (position 1 normal straight, position 2 complete bending and at position 3 again at normal straight position). Hence, we subjected about 75 cycles and surface resistivity was measured at each state. It is worth mentioning that after each cycle the conductive fabric is showing good recovery in resistivity. That means even after 75 cycles there was a minor increase in resistivity, as shown in Figure 18a. Therefore, we can say that developing conductive fabrics are quite stable in the recovery of resistivity values. This behavior is due to the re-establishment of a conductive network of coating, and resuming the initial state of fibres [25]. Furthermore, these stretch and release states are further explained by the expanding first five cycles, as shown in Figure 18b.

The intake of copper in excess of biological needs creates an adverse effect on the human body, including gastrointestinal distress, hemolysis, kidney and liver damage in humans [26]. Therefore, the removal of copper, silver particles and removal of copper coating after electroplating was verified against washing. The electrical surface resistivity of all conductive fabrics was measured before and after washing, as shown in Table 3. There was an insignificant change in electrical conductivity values of conductive fabrics before and after washing. Hence, we can expect the similar behavior for EMI shielding properties after washing. Furthermore, there was insignificant increase in the electrical resistivity of the conductive fabrics was noticed before and after washing. This behavior is attributed due to the efficient metal coating on the surface of cotton fabric without deterioration of electrical conductivity.

**Figure 19:**
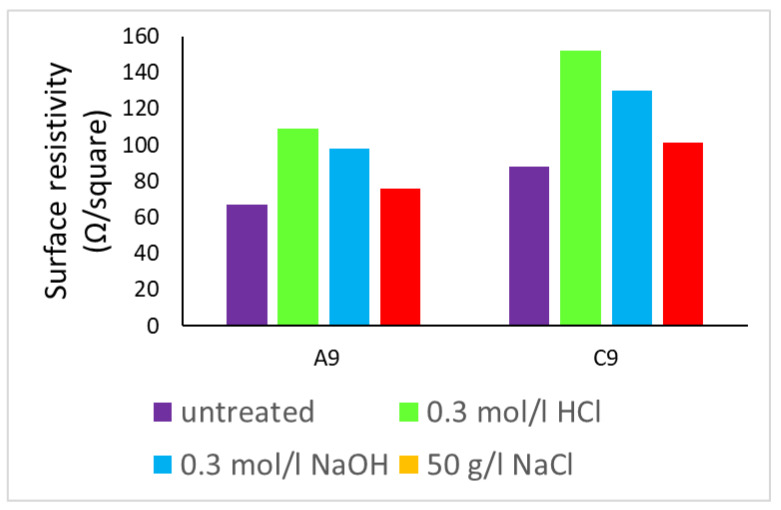
The comparison of electrical resistivity values before and after chemical treatment for Copper electroplated fabrics.

The average surface resistance of copper electroplated samples C9 (copper plating over copper particles coated woven fabric) and A9 (copper plating over silver particles coated woven fabric), was noted after treatment with different types of chemical solutions for the duration of 24 h. Their values of electrical surface resistivity before and after chemical treatment are shown in Figure 19. The insignificant decrease in surface resistivity even after different types of severe chemical treatment reveals the fact that they are electrically conductive.

(Copper electroplated) fabrics can maintain conductive properties in a corrosive environment.

## 4. Conclusions

The study was focused on developing copper electroplated multifunctional fabrics. Before electroplating, the fabrics were activated with the in situ deposition of silver and copper particles. The electrical resistivity for all developed fabric samples was studied at each step (after deposition of particles and after electroplating). The lowest value of electrical resistivity was observed for copper plating over silver coated fabrics. Surface morphology of all conductive fabrics were also studied through SEM, EDX and XRD. Moreover, the electromagnetic shielding and ohmic heating capacity were also analysed. The EMI shielding was found to increase with an increase in the percentage mass gain with plating, which was attributed to increased reflection of EM waves due to a dense, uniform and percolated network of conductive copper coating on the surface. A minimum resistivity of 67 Ω, with 66 dB EMI shielding and 118 °C at 10 V Ohmic heating was observed. Finally, the durability of all developed conductive fabrics was assessed a number of ways (mechanical action, chemical treatment and washing durability). During the mechanical action, the fabrics were subjected to different numbers of bend and release cycles, and electrical conductivity was noted. The electroplated fabric samples showed constancy against electrical resistivity values even after 75 cycles. Similarly, the conductive textiles showed stability against different types of chemical solutions. In the end, the washing stability was also checked, and we observed insignificant loss in electrical conductivity even after intense washing.

## Figures and Tables

**Table 1:** The design of the experiment for electroplating the textile samples.

**Sr. #**	**Sample ID**	**Concentration of Copper Sulphate g/L**	**Time of Exposure (Minutes)**
1	C1	10	10
2	C2		20
3	C3		30
4	C4	20	10
5	C5		20
6	C6		30
7	C7	30	10
8	C8		20
9	C9		30
10	A1		10
11	A2	10	20
12	A3		30
13	A4		10
14	A5	20	20
15	A6		30
16	A7		10
17	A8	30	20
18	A9		30

**Table 2:** Resistivity values of conductive fabrics against concentrations of copper salt.

Sr. #	Sample ID	Concentration of Copper Sulphate g/L	Time of Exposure (Minutes)	Ω/Square
1	C1	10	10	856 ± 32
2	C2		20	456 ± 41
3	C3		30	134 ± 12
4	C4	20	10	567 ± 29
5	C5		20	272 ± 21
6	C6		30	109 ± 9
7	C7	30	10	372 ± 40
8	C8		20	121 ±28
9	C9		30	88 ± 7
10	A1		10	705 ± 32
11	A2	10	20	312 ± 41
12	A3		30	111 ± 12
13	A4		10	423 ± 29
14	A5	20	20	233 ± 23
15	A6		30	109 ± 9
16	A7		10	282 ± 37
17	A8	30	20	99 ± 17
18	A9		30	67 ± 8

**Table 3:** Electrical resistivities of conductive fabrics before and after washing.

Fabric Samples	Electrical Resistivity (Ω/Square)	
	Before Washing	After Washing
Copper particles coated woven fabric	912 ± 26	956 ± 38
Silver particles coated woven fabric	1145 ± 35	1198 ± 47
Copper plating over copper particles coated woven fabric	88 ± 7	97 ± 7
Copper plating over silver particles coated woven fabric	67 ± 8	81 ± 11

## Data Availability

Not applicable.
